# Impact of atopic dermatitis on renal dysfunction: insights from patient data and animal models

**DOI:** 10.3389/fimmu.2025.1558596

**Published:** 2025-03-21

**Authors:** Arisa Ikeda, Ge Peng, Wanchen Zhao, Alafate Abudouwanli, Shigaku Ikeda, François Niyonsaba, Yusuke Suzuki

**Affiliations:** ^1^ Department of Nephrology, Juntendo University Graduate School of Medicine, Tokyo, Japan; ^2^ Atopy (Allergy) Research Center, Juntendo University Graduate School of Medicine, Tokyo, Japan; ^3^ Department of Dermatology and Allergology, Juntendo University Graduate School of Medicine, Tokyo, Japan; ^4^ Faculty of International Liberal Arts, Juntendo University, Tokyo, Japan

**Keywords:** atopic dermatitis (AD), albuminuria, dyslipidemia, inflammation, podocyte, renal dysfunction, S100A calcium-binding protein

## Abstract

**Introduction:**

Atopic dermatitis (AD) is a chronic inflammatory skin disease characterized by pruritus, immune dysregulation, and compromised skin barrier function. Although there are some reports that indicate a link between AD and chronic kidney disease (CKD), the prevalence and underlying mechanism of the association between AD and CKD are still unclear. We aimed to clarify the mechanism underlying the association between AD and CKD using an AD-like mouse model.

**Methods:**

Human serum and urine samples from adults in the U.S. were analyzed using data from the National Health and Nutrition Examination Survey (NHANES). An AD-like mouse model was established by repeatedly applying 2,4-dinitrochlorobenzene to the backs and ears of the mice. Kidney inflammation and podocyte function were evaluated via PAS and H&E staining, immunofluorescence staining, and electron microscopy.

**Results:**

We found that compared to healthy subjects in the NHANES cohort study, patients with AD had altered kidney function. AD-like model mice exhibited albuminuria and renal dysfunction one to three months after the induction of AD. In addition, there were remarkable decreases in triglyceride and very-low-density lipoprotein levels and increases in low-density lipoprotein and non-high-density lipoprotein levels in AD-like model mice. After histological staining of the kidneys of AD-like model mice, macrophage and neutrophil infiltration was detected, and the foot process effacement of podocytes was observed via electron microscopy. In addition, the gene expression of slit diaphragm- and podocyte-related proteins such as nephrin, podocin, and synaptopodin decreased, whereas the gene expression of inflammatory mediators such as S100A8 and S100A9 increased.

**Discussion:**

Following improvements in skin inflammation, alleviation of albuminuria, renal dysfunction and dyslipidemia were observed. These findings suggest that AD-related cutaneous inflammation is associated with albuminuria and podocyte dysfunction.

## 1 Introduction

Chronic kidney disease (CKD) is a global public health threat, as approximately 9.1% of the world’s population suffers from this condition (1). The Kidney Disease Improving Global Outcomes (KDIGO) guidelines define CKD using kidney damage markers, including proteinuria and the glomerular filtration rate (GFR). A GFR less than 60 mL/min and an albumin concentration greater than 30 mg/g, along with abnormalities in kidney structure or function for more than three months, indicate the presence of CKD. End-stage renal disease (ERSD) is defined as a GFR of less than 15 mL/min (2, 3).

Low-grade systemic inflammation is one of the substantial contributors to the development of CKD; however, the exact timeline between the initiation of inflammation and CKD is unclear. Low-grade systemic inflammation involves the constant presence of inflammatory markers. Various conditions, such as metabolic syndrome, nonalcoholic fatty liver disease, cardiovascular disease and diabetes, are associated with low-grade inflammation (4). The inflammatory cascade is characterized by an inciting stimulus related to tissue injury or foreign entities, which increases the generation of proinflammatory cytokines, such as tumor necrosis factor-α and interleukin (IL)-1, resulting in increased blood flow, upregulation of chemical mediators and leukocyte infiltration (5).

Podocytes are highly specialized cells within the glomerulus that are essential for ultrafiltration and express high levels of proteins such as synaptopodin. The glomerulus is the filtration system of the kidney, and the number of glomeruli on one side of the kidney is estimated to be one million. Podocytes form the glomerular filtration barrier, and their foot processes are highly dynamic cellular extensions. The foot processes are connected by slit diaphragm components such as nephrin and podocin, the disruption of which is involved in the development of proteinuria (6).

Atopic dermatitis (AD) is a common chronic relapsing inflammatory skin disease that repeatedly cycles through exacerbation and improvement phases. AD is characterized by pruritus, immune dysregulation, and compromised skin barrier function. AD has a complex etiology involving the interaction of genetic and environmental factors, and people with a family history of AD are at increased risk of developing this condition (7). Skin barrier abnormalities and immune dysfunctions, particularly conditions with a predominance of type 2 helper T (Th2) cytokines, are considered crucial to the pathogenesis of AD (8, 9). In addition, recent reports have suggested a potential relationship between AD and dyslipidemia (10, 11).

A large cohort study of 56,602 CKD patients adjusted for age, sex, habits, diabetes, hypertension, smoking, alcohol use, and obesity reported a higher prevalence of chronic skin conditions such as AD and psoriasis in people with CKD stages 3-5, suggesting that inflammatory skin diseases might be associated with CKD (12). In addition, Kim et al. reported that patients with AD have higher albumin/creatinine ratio values than healthy controls (13), indicating potential renal involvement in AD. Mechanistically, type 2-skewed inflammation, driven by Th2 cytokines, along with the activation of neutrophils and macrophages, plays a pivotal role in the pathogenesis of AD (14). Notably, Th2 cytokines, such as IL-4 and IL-13, as well as neutrophil- and macrophage-mediated inflammation, are also implicated in the progression of renal diseases, contributing to glomerular injury, proteinuria, and chronic kidney inflammation (15–17). These shared inflammatory pathways may underlie the potential pathophysiological link between AD and kidney disease.

However, the relationship between AD and kidney disease and its underlying mechanism are still unknown. Early detection of renal involvement in AD and timely interventions targeting shared inflammatory pathways may help prevent the progression to ESRD. This study aimed to elucidate the potential mechanisms underlying the relationship between AD-related inflammation and altered kidney function, providing new insights into the clinical management of these patients.

## 2 Materials and methods

### 2.1 Study population

This study utilized data collected from 1996–2006 as part of the National Health and Nutrition Examination Survey (NHANES), a nationally representative cross-sectional survey of noninstitutionalized civilian populations in the U.S.A. that is conducted by the National Center for Health Statistics every 2 years (18). The study protocol was approved by the National Center for Health Statistics Research Ethics Review Board, and all participants provided written informed consent. A total of 14,323 participants aged 20 years and older were included in this study. Participants without estimated GFR (eGFR) data, urine albumin–creatinine ratio (UACR) values, or incomplete data on AD and covariates were excluded. Additionally, to avoid the confounding effects of pre-existing kidney disease, hypertension, and diabetes, we excluded patients with impaired kidney function, hypertension, or diabetes from the study. The UACR was calculated as the ratio of urinary albumin to creatinine in spot urine samples. Serum and urinary creatinine were measured via the Jaffe rate method, and urinary albumin was measured via a fluorescence-based immunoassay. The eGFR was estimated using the CKD Epidemiology Collaboration equation based on the serum creatinine level (19). The diagnosis of AD was made according to the ‘Dermatology’ or ‘Allergy’ section of the NHANES and defined based on the following questions: “During the past 12 months, have you had dermatitis, eczema, or any other type of red, inflamed skin rash?” and “Has a doctor ever told you that you have eczema?” The covariates included discrete variables (sex) and continuous variables (age and body mass index).

### 2.2 Establishment of AD-like mouse models

Three types of AD-like mouse models were established in this study. The mice were maintained under specific pathogen-free conditions with a 12-hour light/dark cycle at a consistent temperature of 24 ± 1°C, with unrestricted access to food and water. The animal care and experimental protocols used were approved by the Institutional Animal Care and Use Committee (IACUC) of Juntendo University Graduate School of Medicine (approval number 2024057). All procedures adhered to the 8th edition of the Guide for the Care and Use of Laboratory Animals, ensuring ethical and humane treatment. The reporting of the animal studies complied with the Animal Research: Reporting *In Vivo* Experiments (ARRIVE) guidelines.

Female BALB/c mice aged 10 weeks (Japan SLC Inc., Tokyo, Japan) were sensitized with 2,4-dinitrochlorobenzene (DNCB) (Fujifilm Wako, Osaka, Japan). On the day before application, the dorsal skin of each mouse was shaved. The ears and back of the mice were treated with 1% DNCB once. Starting four days later, 0.4% DNCB was applied at the same site 3 times per week for 12 weeks, and skin and kidney samples were collected on day 90 (**Supplementary Figure 1**). Furthermore, calcipotriol (MC903), a vitamin D3 analog, was topically applied to the shaved dorsal skin of C57BL/6 mice (aged 10 weeks, Japan SLC Inc.) daily for 12 days to induce AD-like dermatitis, and samples were collected on day 13. In addition, AD-like dermatitis was induced in NC/Nga mice (aged 7-8 weeks; Japan SLC Inc.) via topical application of 100 mg of *Dermatophagoides farinae* extract ointment on the shaved dorsal skin twice a week for 3 weeks. Samples were collected on day 22.

### 2.3 Evaluation of albuminuria in mice

The urine of the mice was collected by using mouse urine collection cages (Natsume Seisakusho Co., Ltd., Tokyo, Japan), and the UACR was determined with a DCA Vantage Analyzer (Siemens Healthineers, Bayern, Germany) following the manufacturer’s instructions.

### 2.4 Biochemical analysis

Serum levels of blood urea nitrogen (BUN), creatinine, albumin, uric acid (UA), triglycerides (TG), total cholesterol (T-Chol), and high-density lipoprotein cholesterol (HDL-C) were evaluated using FUJI DRI-CHEM Slides and DRI-CHEM NX-500V (Fujifilm).

### 2.5 Periodic acid–Schiff staining, H&E staining and immunohistochemical staining

Periodic acid–Schiff (PAS) staining, H&E staining and immunohistochemical staining were performed as previously reported (20). Paraffin sections of mouse kidneys (4 μm) were stained with PAS and H&E for morphological analysis. For immunohistochemical analysis, paraffin sections were washed in xylene and hydrated in a series of decreasing alcohol dilutions and water for deparaffinization. Antigen recovery was performed in citrate buffer, pH 6.0, for 40 minutes at 95°C. The sections were blocked using an avidin-biotin blocking kit (Vector Laboratories, Burlingame, CA), followed by incubation with 2% bovine serum albumin (BSA) in phosphate-buffered saline (PBS) for 30 minutes at room temperature, and then incubated overnight at 4°C with the appropriate primary antibodies. The next day, endogenous peroxidase activity in the sections was inactivated with 0.3% hydrogen peroxide/methanol, followed by incubation with secondary antibodies and horseradish peroxidase-conjugated streptavidin. The sections were then stained with 3,3’-diaminobenzidine solution and counterstained with hematoxylin. Images were acquired via optical microscopy. The antibodies used are listed in **Supplementary Table 1**.

### 2.6 Immunofluorescence staining

Immunofluorescence staining of mouse tissues was performed using 4 μm paraffin-embedded kidney sections. After fixation, deparaffinization, and antigen retrieval, the sections were blocked in 2% BSA containing 5% normal goat serum for 30 minutes. The samples were incubated overnight at 4°C with primary antibodies. After washing, Alexa Fluor 594-conjugated goat anti-rabbit IgG (Thermo Fisher Scientific, A11037; 1:1000, Waltham, MA) or Alexa Fluor 488-conjugated goat anti-mouse IgG (Thermo Fisher Scientific, A12379; 1:1000) was added, and the mixture was incubated for 1 hour. The sections were examined using a Zeiss LSM700 instrument equipped with a high-resolution camera (Zeiss, Oberkochen, Germany) and quantitated using Zen 2011 SP3 (black edition) image analysis software (Zeiss). Quantification of the fluorescence intensities of the images was performed with ImageJ software (version 1.52a; National Institutes of Health, Bethesda, MD). On average, 5 randomly selected glomeruli were assessed per mouse. The antibodies used are listed in **Supplementary Table 2**.

### 2.7 Transmission electron microscopy

Transmission electron microscopy analysis was performed as previously reported (21). Briefly, mouse kidneys were fixed overnight at 4°C in 2.5% (vol/vol) glutaraldehyde in 0.1 M PBS (pH 7.4) and postfixed in 1% osmium tetroxide in 0.1 M PBS. The tissues were dehydrated through a graded ethanol series and embedded in Epon 812 (Oken-Shoji, Tokyo, Japan), and ultrathin sections were cut with an ultramicrotome (model UC6, Leica, Wetzlar, Germany) and placed on copper grids. The sections were analyzed using an H-600IV transmission electron microscope (Hitachi, Tokyo, Japan).

### 2.8 Quantitative real-time PCR

Total RNA was extracted from the kidney cortex of each mouse using the RNeasy Plus Universal Mini Kit (QIAGEN, Hilden, Germany) or the RNeasy Plus Micro Kit (QIAGEN). Reverse transcription was performed using ReverTra Ace qPCR RT Master Mix (Toyobo, Osaka, Japan) according to the manufacturer’s instructions. Real-time PCR was performed using the QuantiTect SYBR Green PCR Kit (QIAGEN). Amplification and detection of mRNA were performed using the StepOnePlus Real-Time PCR System (Life Technologies, Carlsbad, CA) following the manufacturer’s specifications. Ribosomal protein S18 (RPS18) was used as the housekeeping gene for normalization in qPCR analysis. The sequences of the primers used in this study are listed in **Supplementary Table 3**.

### 2.9 ELISA

Mouse serum was collected to measure total IgE and S100A8/9 levels. The plates were coated with 2 μg/mL purified rat anti-mouse IgE (553413, BD Biosciences, Franklin Lakes, NJ) overnight at 4°C, followed by blocking with 20% ImmunoBlock at 37°C for 90 minutes. Samples and purified mouse IgE (554118, BD Biosciences) were added to the assay wells and incubated at 37°C for 80 minutes, followed by incubation with horseradish peroxidase–conjugated anti-mouse IgE (LO-ME-2-HRP-1, Dianova, Swiss) and detection with TMB substrate (555214, BD Biosciences). The reaction was stopped with sulfuric acid, and the optical density at 450 nm was read using a plate reader. Serum samples were collected from AD-like mice, and the concentration of S100A8/9 was measured using a DuoSet ELISA kit (R&D Systems, Minneapolis, MN). Briefly, the plates were coated with an S100A8/9 capture antibody overnight at room temperature, followed by blocking with reagent diluent for 60 minutes. After washing, S100A8/9 standards, control samples, and diluted serum samples of AD-like mice were added to the wells and incubated for 2 hours. Following additional washing steps, an S100A8/9 detection antibody was applied, and the plates were incubated again for 2 hours. The reaction was terminated with sulfuric acid, and optical density at 450 nm was measured using a microplate reader.

### 2.10 Statistical analysis

All statistical analyses were performed using GraphPad Prism software (GraphPad Software, version 10.2.0; San Diego, CA). Student’s *t* test was used for comparisons between two groups, whereas one-way analysis of variance (ANOVA) with Tukey’s multiple comparisons test was used for comparisons among multiple groups. p < 0.05 was considered to indicate a statistically significant difference.

## 3 Results

### 3.1 Patients with AD are at risk of renal dysfunction

**Figure 1:**
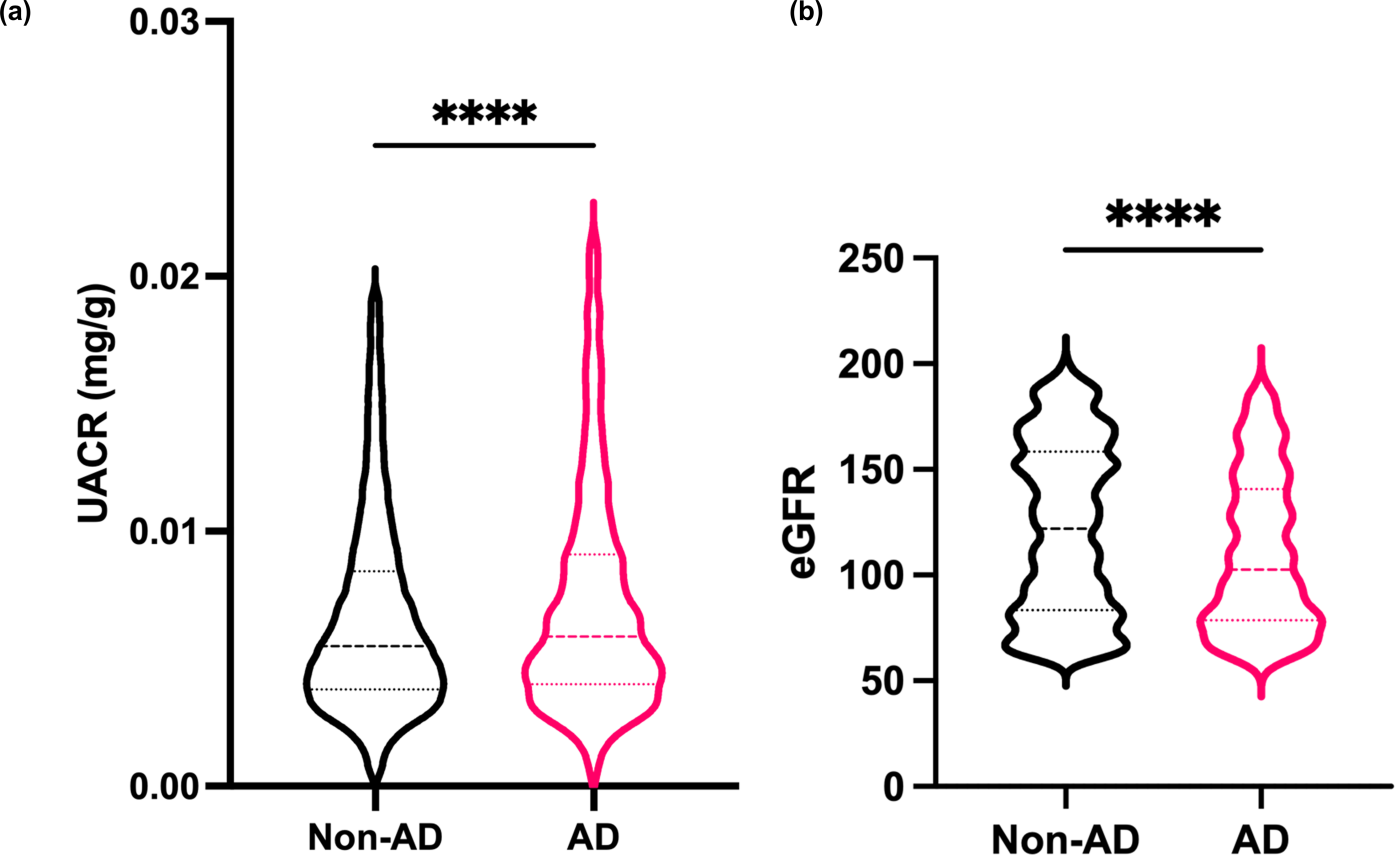
Patients with AD may be at risk of renal dysfunction **(a)** The albumin–creatinine ratio (UACR) was evaluated in urine samples from healthy individuals and AD patients from the NHANES. **(b)** The estimated glomerular filtration rate (eGFR) was evaluated from serum samples from the NHANES. Mean ± SD. ****P<0.0001. Statistical significance was determined by a 2-tailed Student’s *t* test.

To evaluate renal function in individuals with AD, we analyzed serum and urine data from the 1996–2016 NHANES dataset. The characteristics of participants with and without AD are summarized in **Supplementary Table 4**. Because there were no significant differences between the two groups in terms of sex, age or body mass index (BMI), no further subgroup classification was performed. The UACR was significantly higher in patients with AD compared to non-AD participants (AD, n=1488; non-AD, n=12835) (**Figure 1a**). Additionally, the serum eGFR was significantly lower in patients with AD than non-AD participants (AD, n=803; non-AD, n=8357) (**Figure 1b**). These findings indicate that patients with AD are at increased risk of renal dysfunction, as evidenced by increased UACR values and reduced renal filtration capacity (decreased eGFR values).

### 3.2 AD-like model mice exhibit albuminuria, renal dysfunction and dyslipidemia

**Figure 2:**
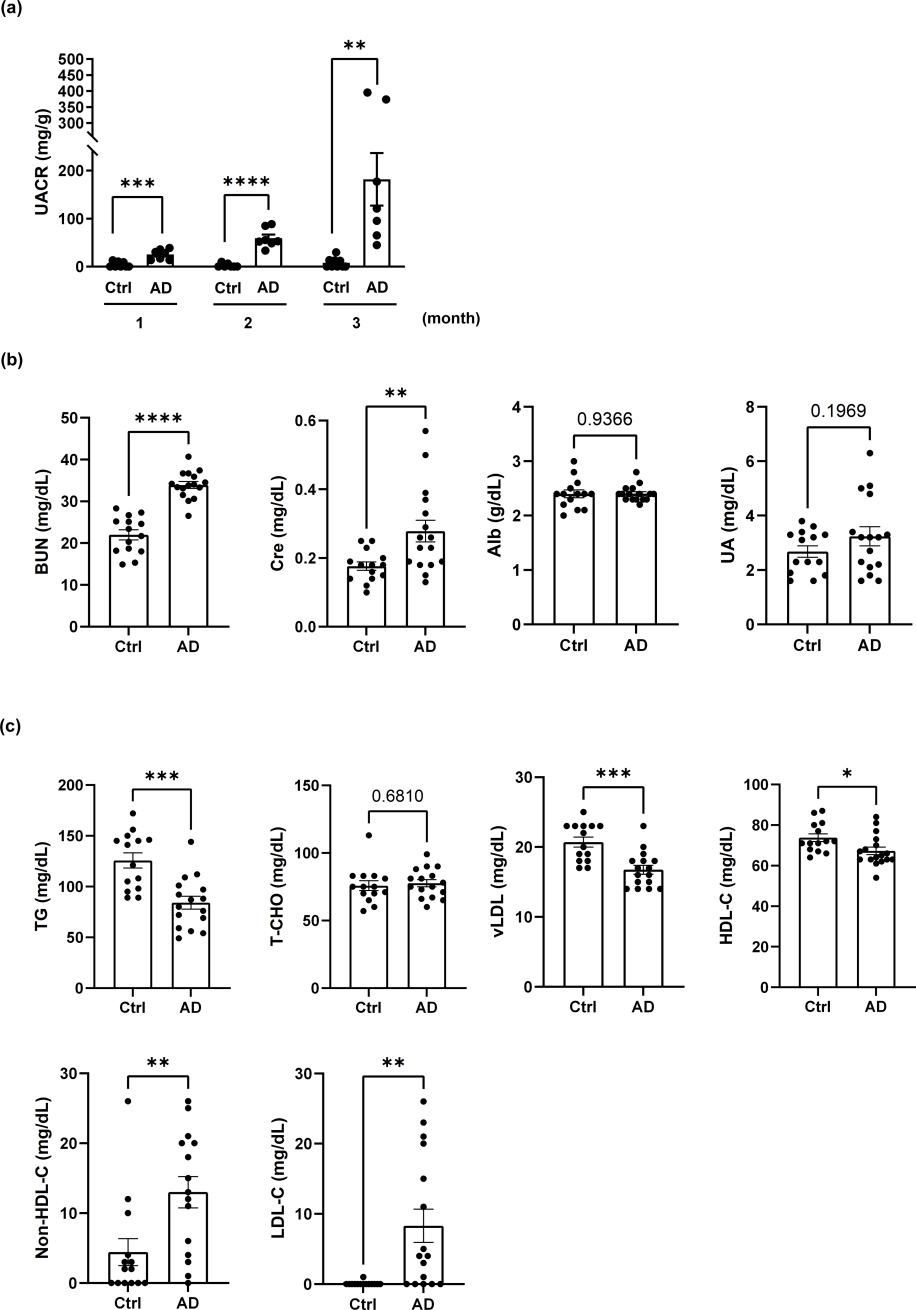
AD-like mice exhibit albuminuria, renal dysfunction and dyslipidemia. **(a)** Changes in the UACR in control mice and mice with DNCB-induced AD-like disease. n = 7–11 per group. Mean ± SD. **(b)** Evaluation of kidney function in control mice and DNCB-induced AD-like model mice. **(c)** Evaluation of dyslipidemia in control mice and DNCB-induced AD-like model mice. n = 14–16 per group. Mean ± SD. *P < 0.05, **P < 0.01, ***P < 0.001, ****P < 0.0001. Statistical significance was determined by a 2-tailed Student’s *t* test.

To further investigate the association of renal dysfunction with AD, we established a DNCB-induced AD-like mouse model. The UACR was significantly higher in DNCB-induced AD-like model mice than in control mice (**Figure 2a**). UACR values were increased in MC903-induced AD model mice (**Supplementary Figure 1a**, left), whereas in *Dermatophagoides farinae* extract-induced NC/Nga AD model mice, the UACR tended to increase, but the difference was not statistically significant (**Supplementary Figure 1a**, right).

Dyslipidemia and renal dysfunction are often closely linked, with dyslipidemia contributing to the progression of renal impairment through mechanisms such as lipid-induced inflammation and endothelial damage (22). In the present study, the serum levels of BUN and creatinine were elevated in DNCB-induced AD-like model mice, indicating impaired renal function, whereas the serum levels of albumin and UA were unchanged (**Figure 2b**). Dyslipidemia characterized by reduced levels of TGs, vLDL and HDL-C, along with increased non-HDL-C and LDL-C levels, was also observed in DNCB-induced AD-like model mice. T-CHO levels were not significantly affected (**Figure 2c**). Similar serum biochemical profiles were observed in MC903-induced and NC/Nga AD model mice (**Supplementary Figures 1b, c**). These findings indicate that AD-like inflammation may be associated with both renal dysfunction and dyslipidemia.

### 3.3 Neutrophils and macrophages infiltrate the kidneys of AD-like model mice

**Figure 3:**
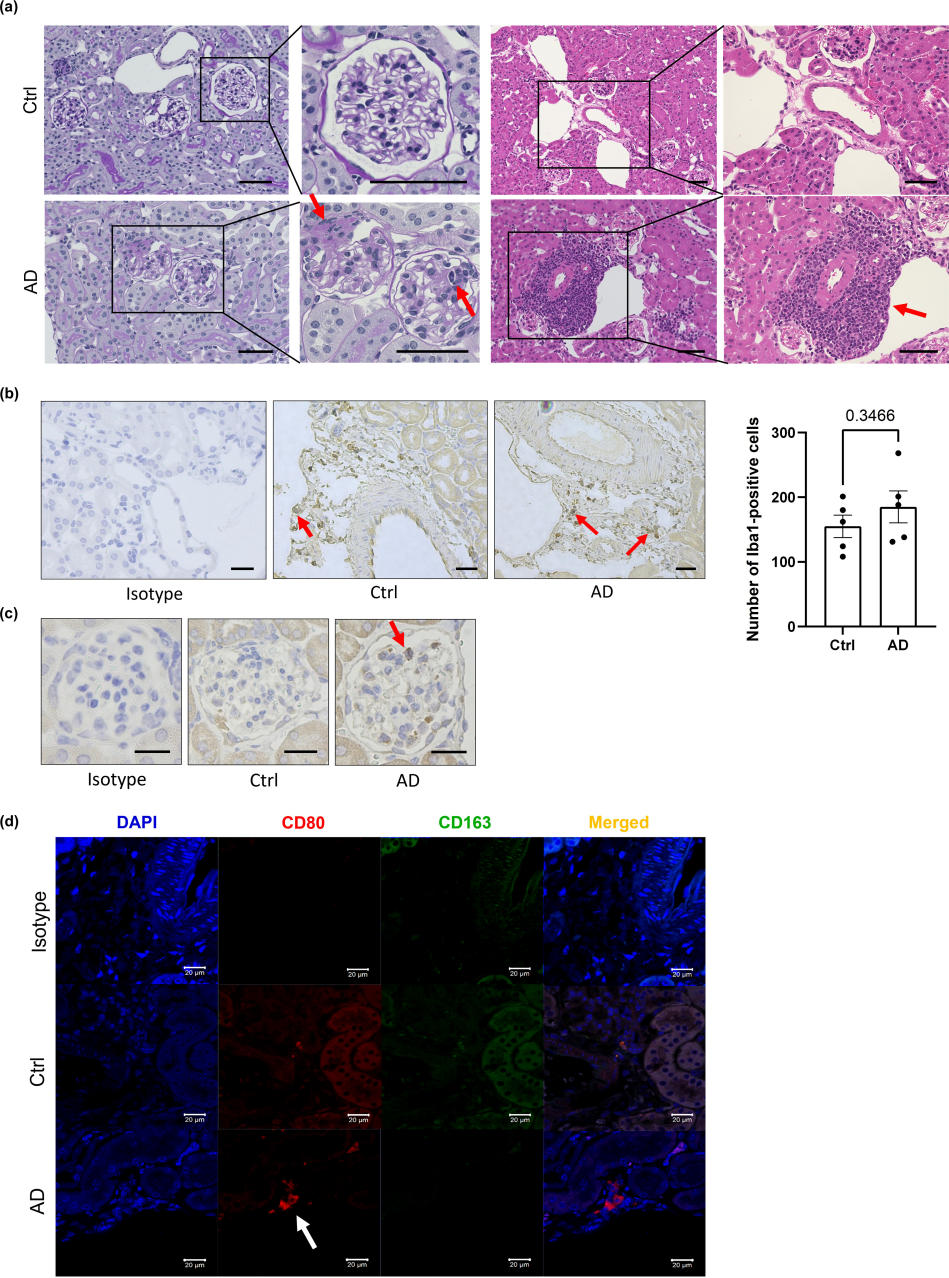
Neutrophils and macrophages may contribute to kidney inflammation in AD-like model mice. **(a)** Representative histological sections of kidneys from control and DNCB-induced AD-like model mice subjected to periodic acid–Schiff (left) and H&E (right) staining. Scale bars: 50 µm. **(b)** Immunostaining of Iba1, a marker of macrophages, in the kidneys of control mice and DNCB-induced AD-like model mice (left) and the number of macrophages (right) in stained sections. Scale bar: 20 µm. **(c)** Immunostaining for MPO, a marker of neutrophils, in the kidneys of control mice and DNCB-induced AD-like model mice. Scale bars: 20 µm. **(d)** Immunofluorescence staining of CD80 and CD163 for the detection of M1 macrophages and M2 macrophages, respectively, in the kidneys of control mice and DNCB-induced AD-like model mice. Scale bars: 20 μm; n = 5 per group. Statistical significance was determined by a 2-tailed Student’s *t* test.

To explore the underlying mechanisms of AD-related renal dysfunction, histological analysis of kidney sections from DNCB-induced AD-like model mice was performed. PAS staining revealed adhesions between the glomerular capillary wall and Bowman’s capsule, as well as inflammatory cell infiltration in some glomeruli, findings commonly associated with acute and chronic glomerulonephritis (**Figure 3a**, left). H&E staining revealed mononuclear cell-dominated inflammatory cell infiltration around the renal vasculature, resembling features observed in acute and chronic tubulointerstitial nephritis (**Figure 3a**, right).

The infiltration of inflammatory cells, including monocytes, macrophages, neutrophils, and T lymphocytes, contributes to the development of kidney injury (23). Immunostaining of kidney sections revealed stronger staining for myeloperoxidase (MPO), a neutrophil marker, and ionized calcium-binding adapter molecule 1 (Iba1), a macrophage marker, in the glomeruli of DNCB-induced AD-like model mice than in those of control mice, although the difference was not statistically significant (P = 0.3466). Neither CD4^+^ nor CD8^+^ T cells were detected (**Figures 3b, c**; **Supplementary Figures 3a, b**). These results suggest that macrophages and neutrophils might be involved in renal inflammation in DNCB-induced AD-like model mice. Similar findings were also obtained in MC903-induced and NC/Nga AD model mice (**Supplementary Figures 3c, d**).

To determine the macrophage subtype involved, kidney sections were stained with CD80 (an M1 macrophage marker) and CD163 (an M2 macrophage marker). Increased numbers of CD80^+^ cells, but not CD163^+^ cells, were observed in DNCB-induced AD-like model mice (**Figure 3d**), indicating a predominance of proinflammatory M1 macrophages during AD-associated renal inflammation.

### 3.4 AD-like chronic inflammation leads to podocyte injury

**Figure 4:**
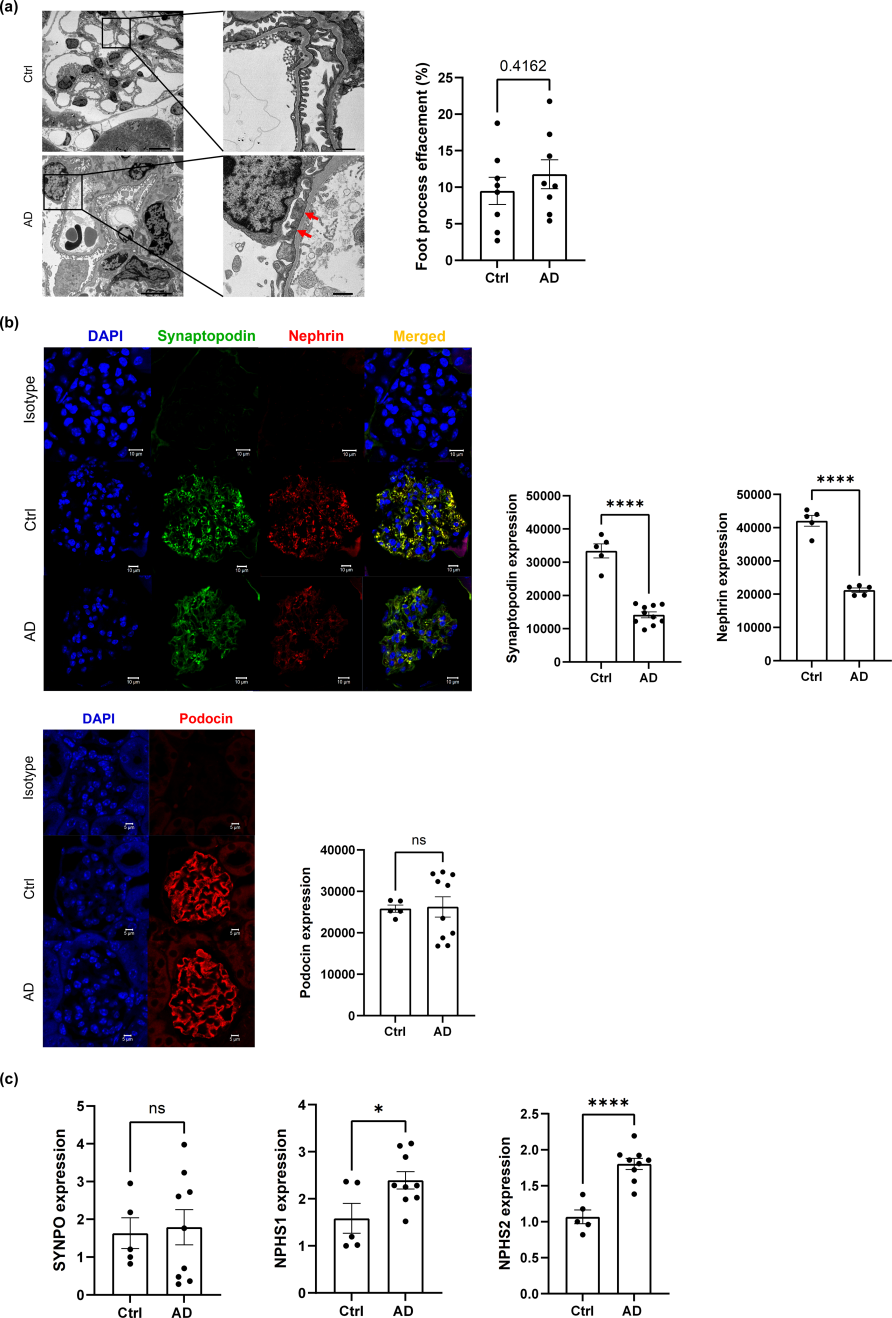
AD-like inflammation may lead to podocyte disorders. **(a)** Representative transmission electron microscopy image of podocytes in glomeruli from control mice and DNCB-induced AD-like model mice (left) and quantification of the percentage of foot process effacement (right). Scale bars: 5 μm (left) and 1 μm (right); n = 8 per group. **(b)** Immunofluorescence staining of synaptopodin, nephrin and podocin was performed in the kidneys of control mice and DNCB-induced AD-like model mice. Scale bars: 10 µm; n =5 per group. **(c)** Real-time PCR analysis of *NPHS1* (nephrin), *NPHS2* (podocin), and *SYNPO* (synaptopodin) in the cortex of the kidney in control mice and DNCB-induced AD-like model mice. n = 6 per group. Mean ± SD. ns, not significant. *P < 0.05, ****P < 0.0001. Statistical significance was determined by a 2-tailed Student’s *t* test. The data are representative of 3 independent experiments.

Since podocyte injury is a critical factor contributing to albuminuria or proteinuria (24), we evaluated the morphological changes in podocytes in DNCB-induced AD-like model mice. Transmission electron microscopy analysis revealed foot process effacement of podocytes in some parts of the glomeruli in DNCB-induced AD-like model mice, indicating podocyte injury. However, there was no significant difference in the foot process effacement rate between DNCB-induced AD-like model mice and control mice (P=0.4162, **Figure 4a**). To further clarify whether there was any damage to the glomeruli in DNCB-induced AD-like model mice, the protein expression of synaptopodin, nephrin and podocin (podocyte-related proteins) was evaluated. Synaptopodin is crucial for maintaining the actin cytoskeleton of podocytes, whereas nephrin and podocin are key components of the slit diaphragm, which is essential for filtration barrier integrity (6). The protein expression of synaptopodin and nephrin decreased in DNCB-induced AD-like model mice (**Figure 4b**, upper panels), whereas there was no change in the protein expression of podocin between the groups (**Figure 4b**, lower panels). Interestingly, quantitative PCR analysis revealed no change in the gene expression of synaptopodin (*SYNPO*), whereas the gene expression of nephrin (*NPHS1*) and podocin (*NPHS2*) significantly increased (**Figure 4c**). These findings suggest that chronic AD-like inflammation affects the structure of podocytes, leading to impaired slit diaphragm integrity and subsequent podocyte injury.

### 3.5 S100A8 and S100A9 expression was increased in the renal cortex of AD-like model mice

**Figure 5:**
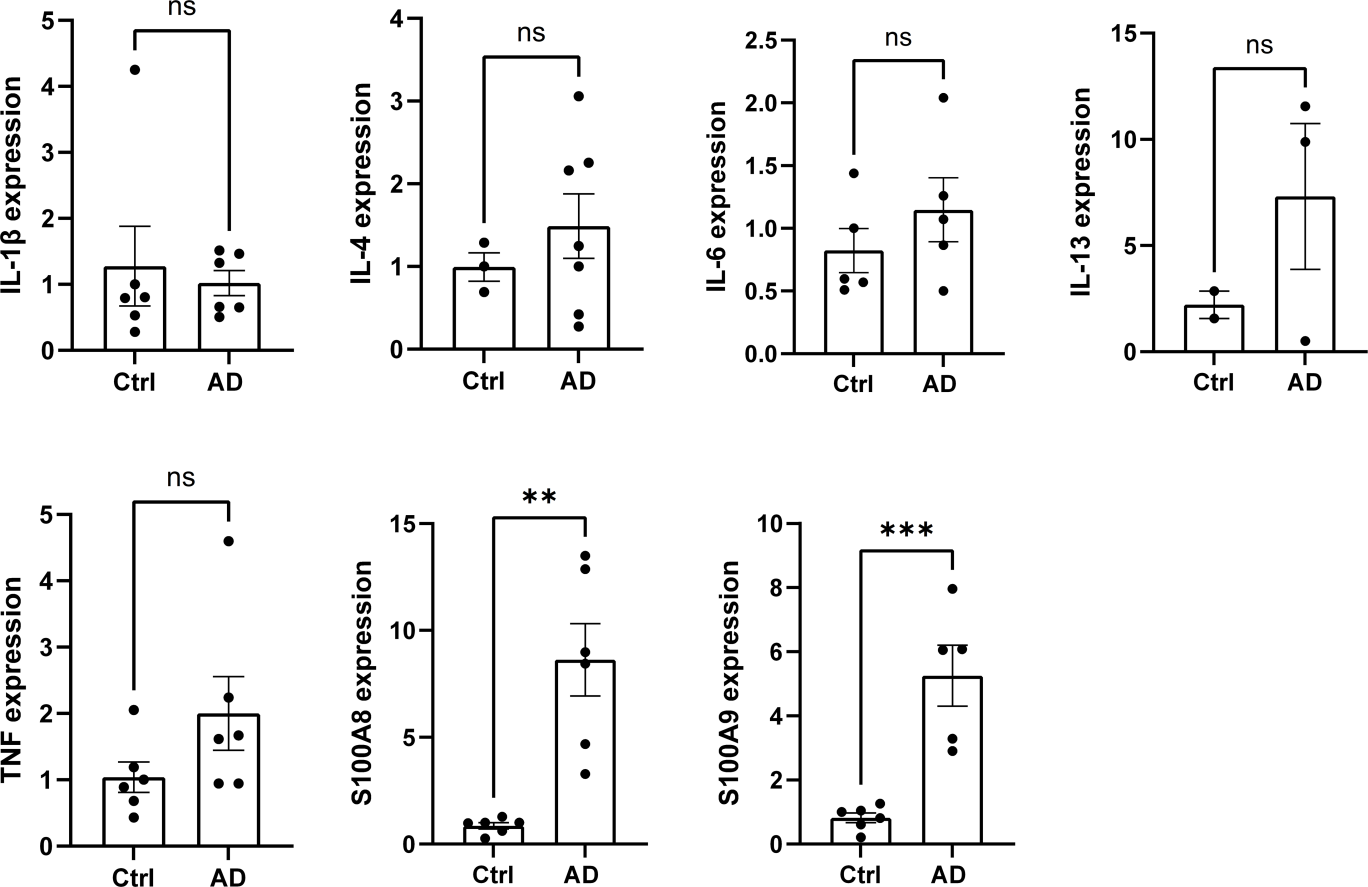
The expression of the inflammatory mediators S100A8 and S100A9 is increased in the renal cortex of AD-like model mice. The mRNA levels of IL-1β, IL-4, IL-6, IL-13, TNF, S100A8, and S100A9 in the cortex of the kidney in control mice and AD-like model mice were measured via real-time PCR. n = 6 per group. Mean ± SD. ns, not significant. **P < 0.01, ***P < 0.001. Statistical significance was determined by a 2-tailed Student’s *t* test. The data are representative of 3 independent experiments.

Given the involvement of neutrophils and M1 macrophages in renal inflammation, we examined the gene expression of inflammatory mediators in the renal cortex of DNCB-induced AD-like model mice. While no significant changes were observed in the gene expression of *IL-1β*, *IL-4*, *IL-6*, and *IL-13* levels, we found a substantial upregulation of S100A8 and S100A9 gene expression in the renal cortex of DNCB-induced AD-like model mice, both of which are associated with neutrophils and M1 macrophages (**Figure 5**). Consistently, no significant changes were found in MC903-induced AD-like model mice or NC/Nga AD-like model mice compared with their respective controls (**Supplementary Figures 4a, b**); however, the serum S100A8/9 levels in these mice were significantly increased (**Supplementary Figure 4c** left (MC903), 4c right (NC/Nga)). These findings indicate that S100A8 and S100A9 may be involved in kidney dysfunction in AD-like mice.

### 3.6 Recovery from inflammation improves renal dysfunction and dyslipidemia in AD-like model mice

**Figure 6:**
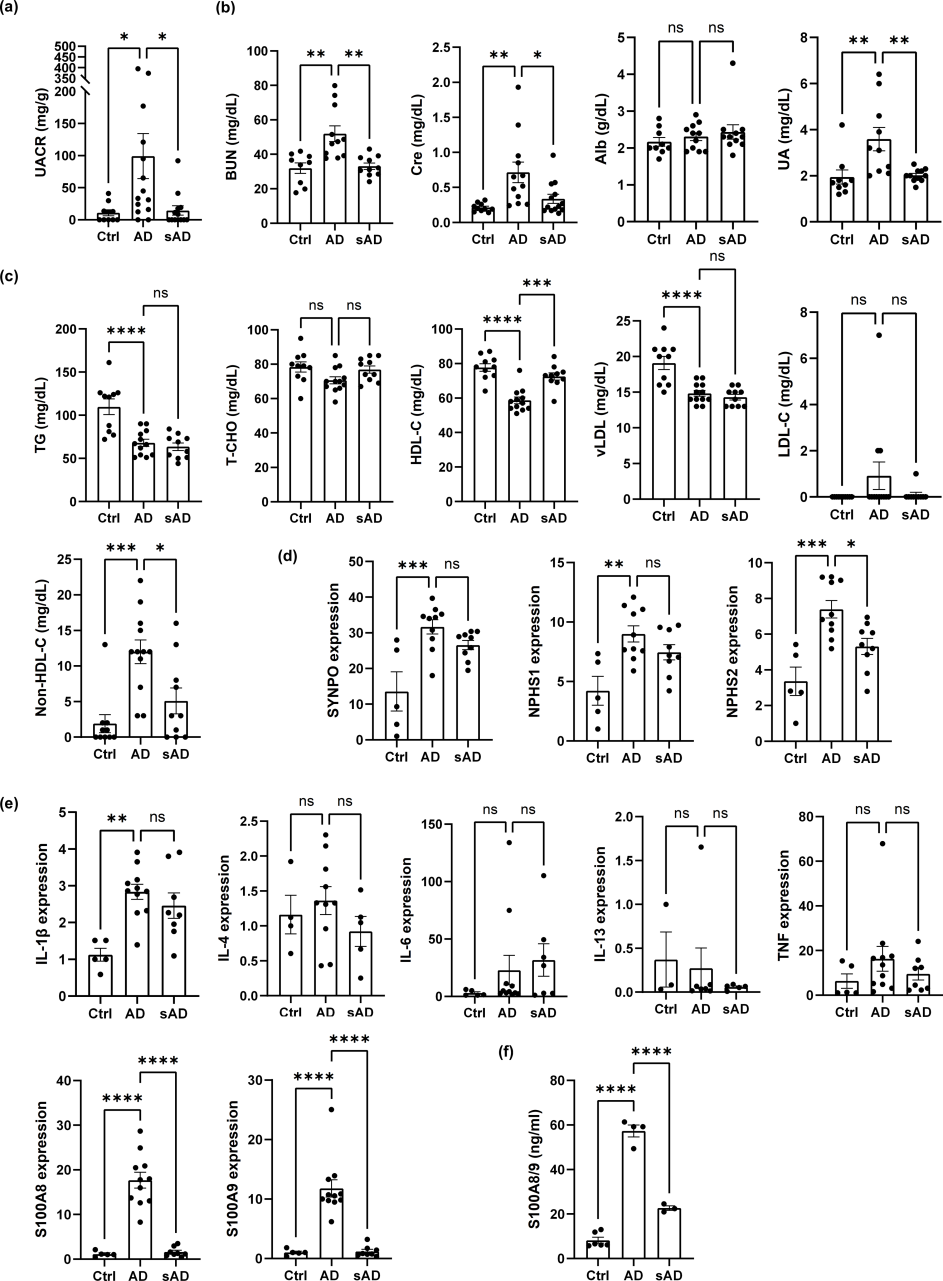
Controlling inflammation improves renal dysfunction and dyslipidemia in AD-like model mice. **(a)** UACR values in DNCB-induced AD-like model mice and sAD model mice. n = 10-15. **(b)** Evaluation of kidney function in control mice and DNCB-induced AD-like model mice and sAD model mice. **(c)** Evaluation of dyslipidemia in control mice and DNCB-induced AD-like model mice and sAD model mice. n = 9–12 per group. **(d)** The mRNA levels of SYNPO, NPHS1, and NPHS2 in the cortex of the kidney in control mice, DNCB-induced AD-like model mice and sAD model mice were measured via real-time PCR. n = 5–11 per group. **(e)** The mRNA levels of IL-1β, IL-4, IL-6, IL-13, TNF, S100A8, and S100A9 in the cortex of the kidney in control mice and DNCB-induced AD-like model mice and sAD model mice were measured via real-time PCR. n = 5–11 per group. **(f)** ELISA to determine the concentrations of the indicated factors in the serum of control mice, DNCB-induced AD-like model mice and sAD model mice. n = 3–6 per group. Mean ± SD. *P < 0.05, **P < 0.01, ***P < 0.001, ****P < 0.0001. Statistical significance was determined by one-way ANOVA with Tukey’s multiple-comparison test. The data are representative of 3 independent experiments.

To determine whether improvement of AD-like inflammation could result in recovery of renal function, DNCB-induced AD-like model mice were divided into two groups: one group with continuous AD induction for 3 months (AD) and another group with AD induction for 2 months followed by cessation of induction for 1 month (subsided AD, sAD). sAD model mice presented a marked improvement in albuminuria (**Figure 6a**). Similarly, the serum BUN, creatinine and UA levels were lower in sAD model mice than in DNCB-induced AD-like model mice, suggesting improved renal function. However, albumin levels remained unchanged (**Figure 6b**).

With respect to dyslipidemia, cessation of AD induction for 1 month resulted in an increase in HDL-C and a reduction in non-HDL-C levels, while the levels of TGs, T-CHO, vLDL and LDL-C did not differ from those in DNCB-induced AD-like model mice or in mice that underwent cessation of AD induction for 1 month (**Figure 6c**). Importantly, the cessation of AD induction also led to a reduction in the gene expression of nephrin (*NPHS1*), podocin (*NPHS2*) and synaptopodin (*SYNPO*), although only the gene expression of podocin was significantly reduced (**Figure 6d**). In addition, among the various inflammatory mediators investigated, only S100A8 and S100A9 exhibited decreased gene expression in the kidney cortex. In contrast, no marked changes were observed in the gene expression of IL-1β, IL-6, IL-4, or IL-13 (**Figure 6e**). We confirmed that the protein levels of S100A8 and S100A9 (S100A8/9) in the serum of DNCB-induced AD-like model mice were significantly elevated and that cessation of AD induction resulted in marked decreases, as assessed by ELISA (**Figure 6f**). These results indicate that improvements in AD-like inflammation can ameliorate renal dysfunction and dyslipidemia in AD-like model mice, potentially by downregulating the gene expression of the inflammatory mediators S100A8 and S100A9.

## 4 Discussion

A cohort study by Schonmann et al. described the association between CKD and chronic inflammatory diseases (12), whereas Wei et al. reported an association between IgA vasculitis and AD in children (25), suggesting an association between chronic inflammatory diseases such as AD and kidney diseases.

The pathogenesis of AD involves various inflammatory factors, with type 2 skewed inflammation being one of the major pathogenic factors of AD (14). Th2 cytokines such as IL-4 and IL-13 are involved in renal diseases (podocyte disorders) that cause proteinuria, such as minimal change nephrotic syndrome (MCNS) (15–17). Therefore, in this study, we hypothesized that inflammatory mediators, including Th2 cytokines associated with AD, may contribute to kidney inflammation.

Our analysis of data from the NHANES (18) revealed an increase in the UACR and a decrease in the eGFR in patients with AD. These results were also confirmed in AD-like mouse models, such as the DNCB-induced AD-like model, MC903-induced AD-like model and NC/Nga AD-like model. We used a model of DNCB-induced AD-like disease in BALB/c mice, a model with an easy-to-induce Th2 immune response that is widely used in allergy studies (26). Notably, both DNCB-induced AD-like model mice and MC903-induced AD-like model mice presented increased albuminuria, and NC/Nga AD-like model mice tended to have increased albuminuria, although the increase was not significant. NC/Nga control mice (without the application of *Dermatophagoides farinae* extract), which are genetically mutant mice, may be more prone to kidney inflammation than normal control mice. In fact, these NC/Nga control mice presented slight skin inflammation and more albuminuria than normal control mice did. This might explain why there was no significant difference in albuminuria between NC/Nga AD-like model mice and NC/Nga controls.

Albuminuria occurs following damage to podocytes, which are kidney-specific cells related to glomerular filtration (27). Crosstalk between podocytes and neutrophils reportedly contributes to disruption of the glomerular filtration barrier in the context of acute glomerulonephritis (28). Furthermore, the activation of macrophages during nephritis in diabetic kidneys promotes renal inflammation and fibrosis in the glomeruli and tubulointerstitium (29). Therefore, the infiltration of neutrophils and macrophages is a key event in kidney inflammation. Among M1 and M2 macrophages, M1 macrophages are proinflammatory; they produce proinflammatory factors such as IL-6 and IL-12 and promote tumor necrosis-α expression. In contrast, M2 macrophages drive anti-inflammatory responses and contribute to the repair of damaged tissues (30). In this study, M1 macrophages were likely dominant in AD-like model mice. In the context of active glomerulonephritis, such as rapidly progressive glomerulonephritis, infiltrating macrophages have a dominant M1 proinflammatory phenotype, and M1 polarization can be triggered by lipopolysaccharide and S100A9 to produce a range of proinflammatory molecules (31). In this study, the glomeruli of kidneys from AD-like model mice contained a few myeloperoxidase-positive cells, suggesting that neutrophils may contribute to kidney inflammation to some extent in AD-like model mice. In contrast, T cells were not detected in the kidneys of AD-like model mice, although these cells are known to promote renal injury in the context of adriamycin nephropathy (32) and renal ischemia-reperfusion injury (33).

In AD-like model mice, the TG, vLDL and HDL-C levels were decreased, whereas LDL and non-HDL levels were increased. Although nephrosis is usually complicated by dyslipidemia and may cause weight gain due to edema, the body weight of AD-like model mice used in our study tended to decrease compared with control mice. This implies that AD-like mice did not develop nephrosis (**Supplementary Figure 5**).

Our study demonstrates that among the inflammatory mediators assessed, S100A8 and S100A9 were significantly upregulated in the renal cortex of AD-like model mice, whereas Th2 cytokines (IL-4 and IL-13) did not show notable changes. This suggests that AD-related kidney inflammation is predominantly mediated by innate immune activation rather than classical Th2 pathway. It has been reported that serum S100A8 and S100A9 levels are increased in AD patients (34). Moreover, S100A8 and S100A9 are Ca^2+^-binding proteins that belong to the S100 family and are constitutively expressed in neutrophils and monocytes as Ca^2+^ sensors, participating in cytoskeletal rearrangement and arachidonic acid metabolism. During inflammation, S100A8 and S100A9 are released and play critical roles in modulating the inflammatory response by stimulating leukocyte recruitment and inducing cytokine secretion (35). S100A8 and S100A9, which are predominantly released by activated neutrophils and macrophages, act as damage-associated molecular patterns (DAMPs) that stimulate toll-like receptor (TLR) 4 signaling on endothelial and immune cells, leading to nuclear factor-kB (NF-κB) activation and enhanced cytokine secretion. This signaling cascade promotes the upregulation of endothelial adhesion molecules, including intercellular adhesion molecule-1 (ICAM-1), vascular cell adhesion molecule-1 (VCAM-1), E-selectin, and P-selectin, which facilitate leukocyte recruitment from circulation into the kidney (36–38). Additionally, S100A8 and S100A9 have been shown to induce neutrophil extracellular trap (NET) formation, a process that contributes to glomerular damage and proteinuria (39–41). NETs are implicated in various kidney diseases, including lupus nephritis and ANCA-associated glomerulonephritis, suggesting a potential role in AD-related renal dysfunction (41). Furthermore, S100A8 and S100A9 signaling in macrophages can promote a shift towards the M1 proinflammatory phenotype, as evidenced by increased CD80^+^ macrophages in the renal cortex of AD-like model mice. Collectively, these findings provide a novel link between AD-associated chronic inflammation and kidney dysfunction through neutrophil and macrophage activation, and highlight S100A8 and S100A9 as potential biomarkers for AD-related renal involvement.

Cessation of AD induction resulted in S100A8 and S100A9 downregulation and improved dyslipidemia. In fact, increases in HDL-C, decreases in non-HDL-C, and improvements in albuminuria and creatinine levels were observed in mice when the induction of AD was discontinued. These observations indicate that alleviation of inflammation in chronic skin conditions such as AD leads to not only the downregulation of the gene expression of inflammatory mediators but also improvements in dyslipidemia, albuminuria and serum creatinine levels. Some cohort studies have reported that patients with asthma and AD have no changes in HDL levels (10), but decreased LDL levels have been reported in pediatric asthma patients (11). In addition, increased levels of the serum S100A8 and S100A9 have been reported in diseases such as acute coronary syndrome, coronary artery calcification, cardiovascular intimal hyperplasia, and atherosclerosis (42), which have a strong association with dyslipidemia. Systemic inflammation is a key contributor to metabolic dysregulation, including dyslipidemia, in chronic inflammatory diseases such as AD and psoriasis (43, 44). In our study, AD-like model mice exhibited elevated serum S100A8 and S100A9 levels along with dyslipidemia, suggesting an interplay between inflammation and lipid metabolism. S100A8 and S100A9 function as DAMPs that activate TLR4 signaling, inducing NF-κB activation and amplifying inflammatory responses. This persistent inflammation may lead to oxidative stress and endothelial dysfunction, which impair lipid homeostasis. Studies have shown that S100A8- and S100A9-mediated inflammation can alter hepatic lipid metabolism by increasing lipogenesis and decreasing cholesterol efflux, contributing to dyslipidemia (45, 46). Moreover, S100A8 and S100A9 may promote monocyte recruitment to vascular endothelium, accelerating atherosclerotic plaque formation (47). These findings align with epidemiological studies indicating that patients with AD have an increased risk of cardiovascular disease (48), partly due to chronic inflammation-induced lipid abnormalities. Our results suggest that the AD-like inflammatory state in our model not only impacts the skin and kidneys but also contributes to systemic metabolic dysfunction. Further research is needed to explore whether targeting S100A8 and S100A9 could mitigate dyslipidemia and reduce cardiovascular risk in patients with AD.

Slit diaphragm-related proteins such as nephrin, podocin and synaptopodin are reduced in pathological conditions such as nephrosis, in which proteinuria is apparent (17). We found that the protein expression of nephrin, podocin and synaptopodin was reduced in AD-like model mice. In contrast, the gene expression of these proteins was elevated in the renal cortex of AD-like model mice, and cessation of AD induction resulted in their normalization. The expression of podocyte-related genes has been reported to be reduced in nephrosis (49). The elevated gene expression of nephrin, podocin and synaptopodin may be a compensatory response to a subtle decrease in these molecules, as microalbuminuria in this study might be due to slight changes in slit diaphragm component levels.

The renal histopathology observed in AD-like model mice shares features with various kidney diseases, including glomerulonephritis and tubulointerstitial nephritis, but does not fully align with any single known kidney disease. The presence of podocyte foot process effacement and slit diaphragm disruption suggests similarities to podocytopathies such as minimal change disease or early-stage focal segmental glomerulosclerosis, both of which are associated with proteinuria. However, unlike these conditions, which sometimes involve podocyte-targeting autoantibodies or T cell dysfunction, the absence of significant T cell infiltration in AD-like model mice suggests an alternative inflammatory mechanism. The observed macrophage and neutrophil infiltration, along with S100A8 and S100A9 upregulation, resembles patterns observed in various kidney diseases, including lupus nephritis, ANCA-associated glomerulonephritis, and IgA nephropathy, where innate immune activation plays a role in disease progression. However, the lack of immune complex deposition and significant glomerular crescents distinguishes the AD-associated kidney pathology from classic autoimmune glomerulonephritis. Taken together, these findings suggest that AD-associated renal dysfunction represents some kinds of inflammatory process, possibly driven by chronic allergic inflammation. Further studies are needed to clarify whether this pattern of kidney involvement is unique to AD or a broader feature of allergic inflammation-related nephropathy.

In conclusion, this is the first experimental study to report a relationship between albuminuria and AD. Our study suggests that the overexpression of inflammatory mediators such as S100A8 and S100A9 during inflammation may contribute to the development of kidney dysfunction. Despite the strengths of this study, several limitations should be acknowledged. First, the diagnosis of AD was based on a self-reported questionnaire rather than physician-confirmed diagnostic criteria. This method may lead to misclassification or underreporting, as individuals with other chronic inflammatory skin conditions could be included in the AD group. Future studies should utilize clinically confirmed AD cohorts to improve diagnostic accuracy. Second, the cross-sectional nature of this study prevents causal inferences between AD and renal dysfunction. While our findings indicate that individuals with AD exhibit higher UACR and lower eGFR, it remains unclear whether AD directly contributes to kidney dysfunction or if both conditions share overlapping inflammatory mechanisms. Longitudinal studies are needed to determine the directionality of this association. Third, while we observed elevated systemic S100A8/S100A9 levels in AD-like mice with renal dysfunction, our study does not establish direct mechanistic links between these inflammatory mediators and kidney injury. Future research should incorporate *in vivo* AD models and *in vitro* experiments to investigate whether S100A8- and S100A9-mediated inflammation directly induces renal damage.

In clinical practice, urinalysis in patients with AD is rare. Therefore, if proteinuria is detected in a patient with AD, it is necessary to consider the latent renal dysfunction associated with chronic skin allergic inflammation. In addition, in the treatment of proteinuria, clinicians should consider renal dysfunction associated with chronic skin inflammation, such as AD, as a differential diagnosis. Our study provides new insights for not only AD guidelines but also albuminuria guidelines.

## Data Availability

The original contributions presented in the study are included in the article/**Supplementary Material**. Further inquiries can be directed to the corresponding authors.
