# Supplementary material for: Impact of atopic dermatitis on renal dysfunction: insights from patient data and animal models
Source: Front Immunol. 2025 Mar 21;16:1558596. doi: 10.3389/fimmu.2025.1558596 (PMC11968387; doi:10.3389/fimmu.2025.1558596)
Supplement: Supplementary file 1 [file DataSheet1.pdf]

# *Supplementary Material*

## Supplementary Figures

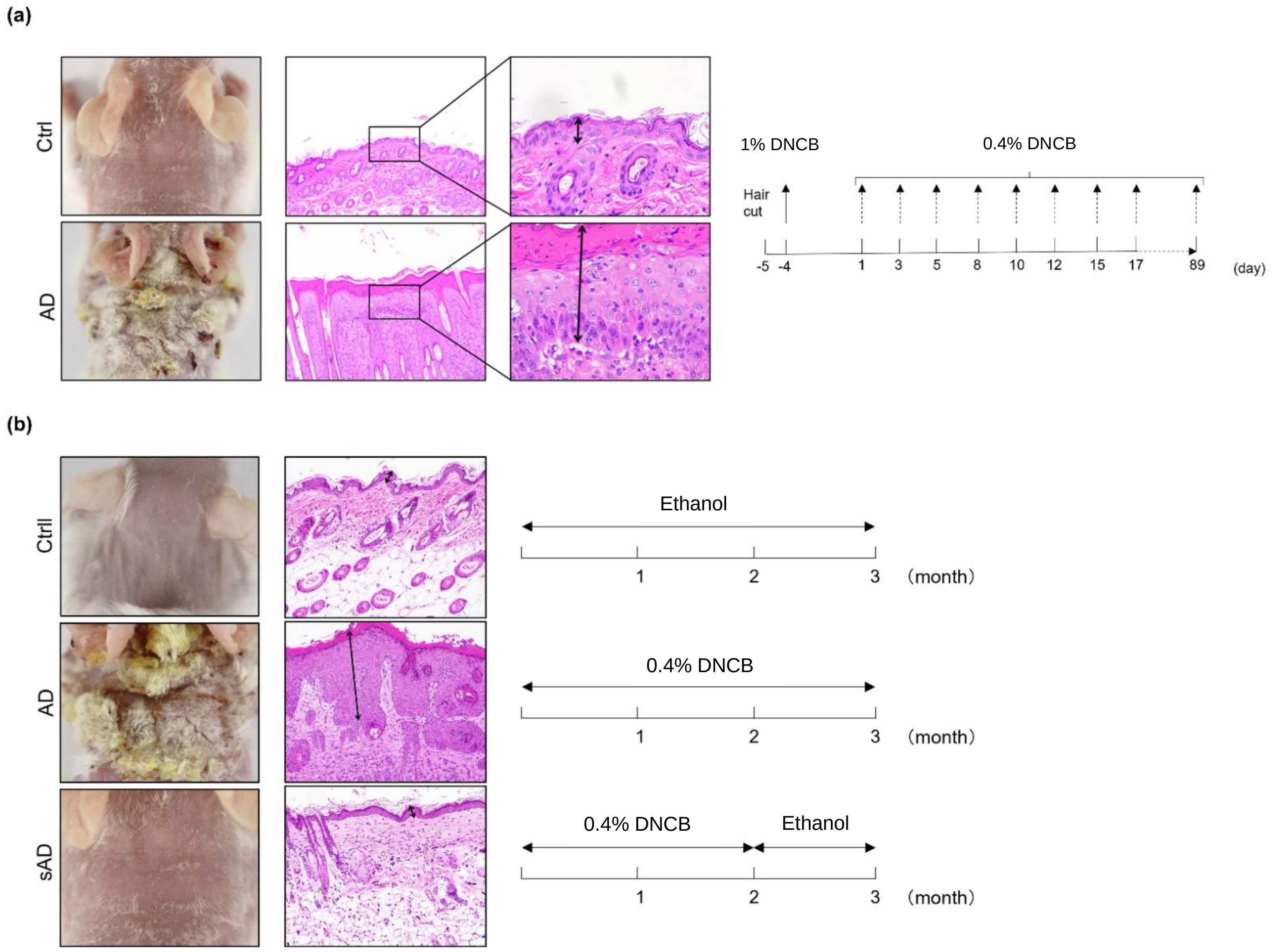


## Supplementary Figure 1

(a) Representative histological sections of the dorsal skin of control mice and DNCB-induced AD model mice after staining with H&E. Magnification: 20× (left) and 40× (right). Ethanol was applied to control mice (Ctrl) 3 times a week for 3 months. DNCB-induced AD model mice were established by applying 0.4% DNCB to the mice 3 times a week for 3 months. (b) Representative histological sections of the dorsal skin of control mice and DNCB-induced AD model mice and sAD model mice after staining with H&E. Magnification: 20×. Ethanol was applied to control mice (Ctrl) 3 times a week for 3 months. DNCB-induced AD model mice (AD) were established by applying 0.4% DNCB 3 times a week for 3 months. DNCB-induced AD model mice in which inflammation subsided (sAD) were established by applying 0.4% DNCB to the mice 3 times a week for 2 months and then administering ethanol 3 times a week for 1 month.

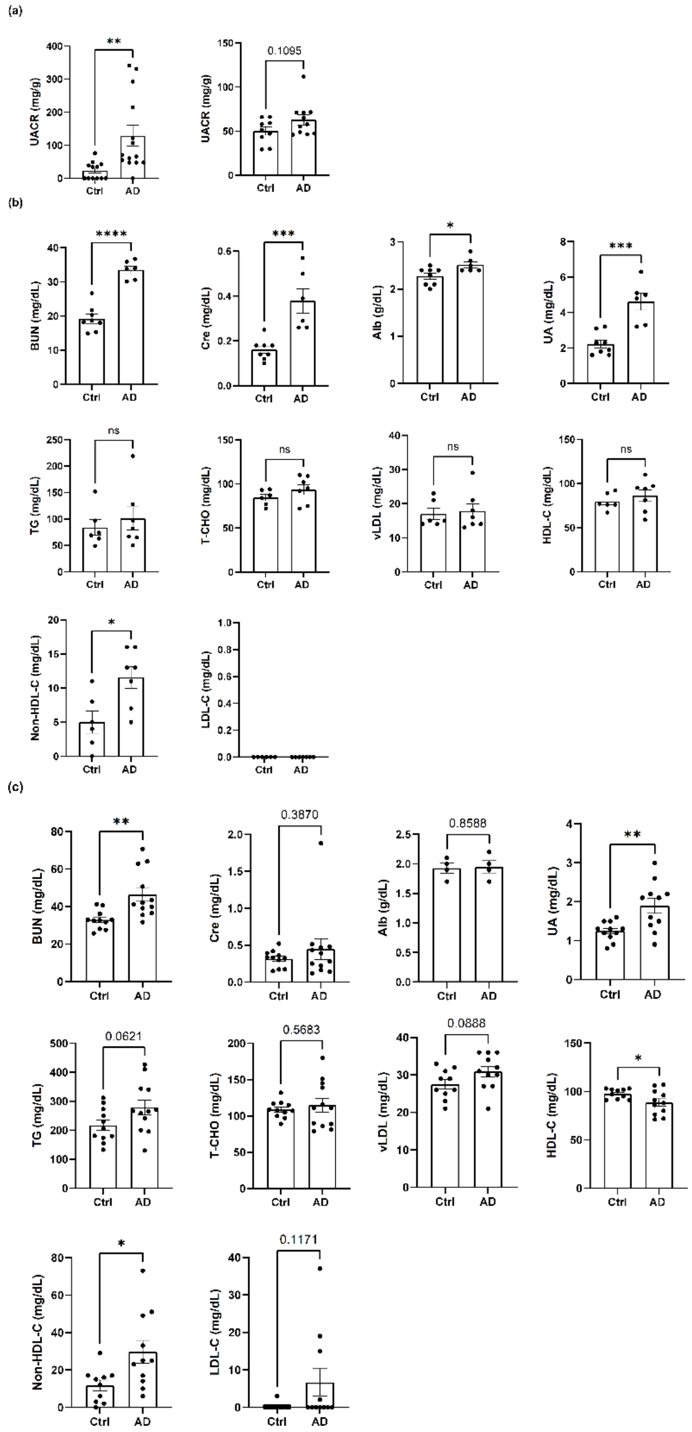


## Supplementary Figure 2

(a) UACR values of control mice and MC903-induced AD-like model mice (left). UACR values of NC/Nga control mice and NC/Nga AD-like model mice (right). n = 7–11 per group. Mean ± SD. (b) Evaluation of kidney function and dyslipidemia in control mice and MC903-induced AD-like model mice. (c) Evaluation of kidney function and dyslipidemia in NC/Nga control mice and NC/Nga AD-like model mice. n = 14–16 per group. Mean ± SD. *P < 0.05, **P < 0.01, ***P < 0.001, ****P < 0.0001. Statistical significance was determined by a 2-tailed Student's *t* test.

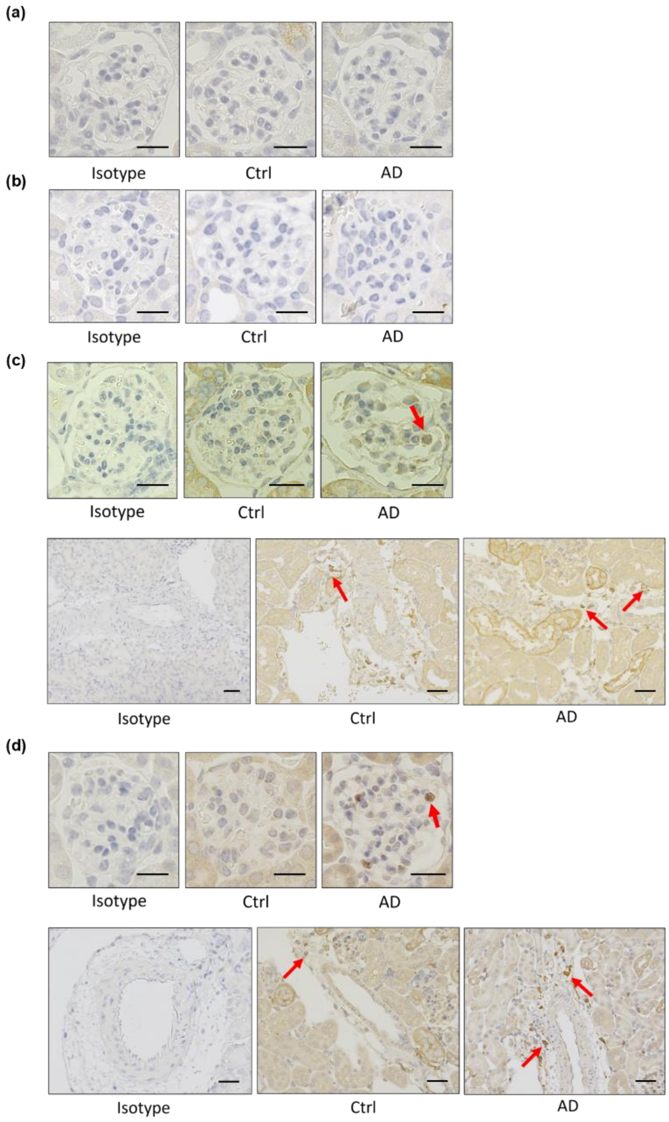


**Supplementary Figure 3**

(a) Immunostaining for CD4 in the kidneys of control mice and DNCB-induced AD-like model mice. Scale bar: 20 µm. (b) Immunostaining for CD8 in the kidneys of control mice and DNCB-induced AD-like model mice. Scale bar: 20 µm. (c) Immunostaining for Iba1 (upper panel) and MPO (lower panel) in the kidneys of control mice and MC903-induced AD-like model mice. Scale bar: 20 µm. (d) Immunostaining for Iba1 (upper panel) and MPO (lower panel) in the kidneys of NC/Nga control mice and NC/Nga AD-like model mice. Scale bar: 20 µm.

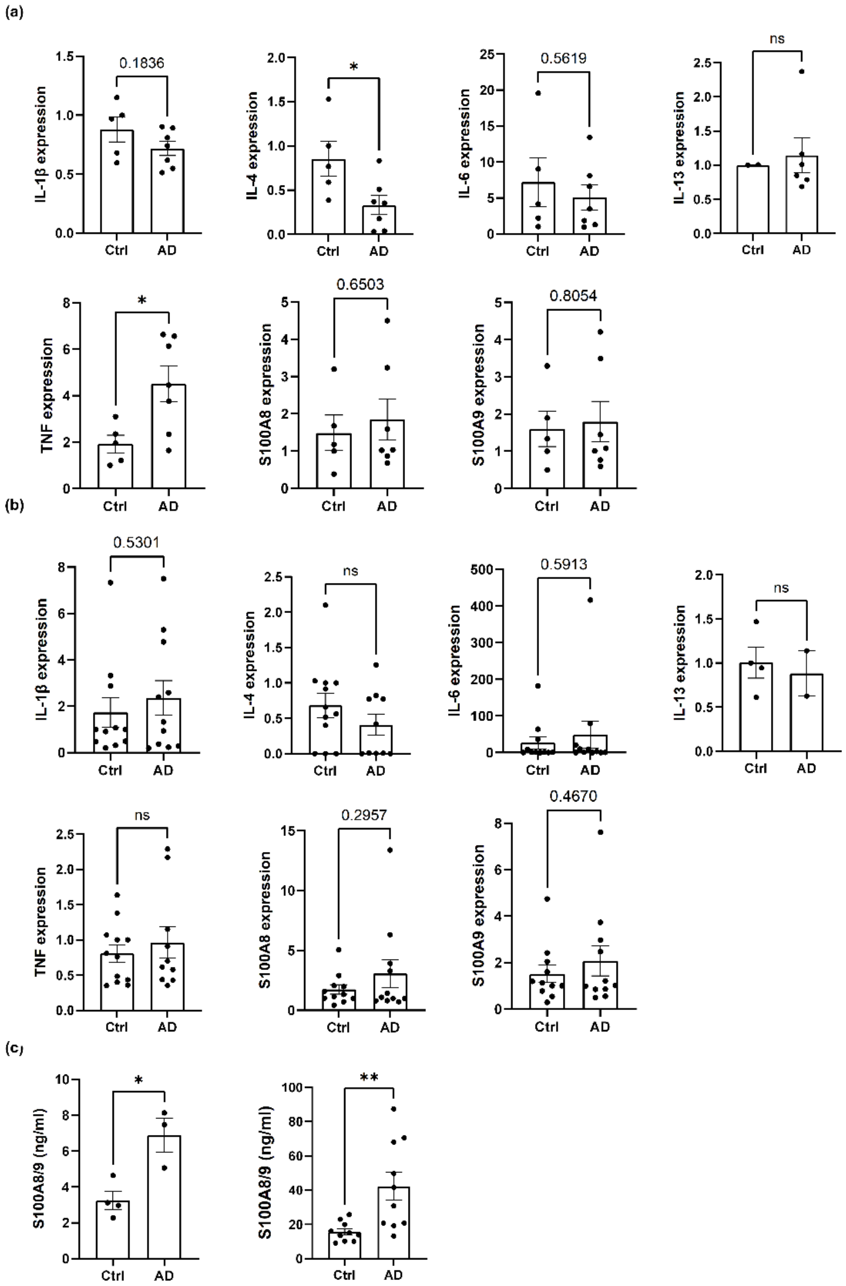


## Supplementary Figure 4

(a) The mRNA levels of IL-1β, IL-4, IL-6, IL-13, TNF, S100A8, and S100A9 in the cortex of the kidney in control mice and MC903-induced AD-like model mice were measured by real-time PCR. n = 5–7 per group. (b) The mRNA levels of IL-1β, IL-4, IL-6, IL-13, TNF-α, S100A8, and S100A9 in the cortex of the kidney in NC/Nga control mice and NC/Nga AD-like model mice were measured by real-time PCR. n= 11 per group. (c) ELISA to determine the concentrations of the indicated factors in the serum of MC903-induced AD-like model mice (left) and NC/Nga AD-like model mice (right). n = 3–10 per group. Mean ± SD. *P < 0.05, **P < 0.01, ***P < 0.001, ****P < 0.0001. Statistical significance was determined by a 2-tailed Student's *t* test. The data are representative of 3 independent experiments.

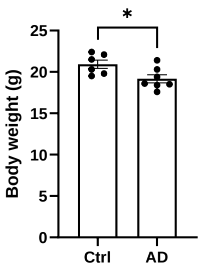


**Supplementary Figure 5**

Body weights of the control and DNCB-induced AD-like model mice. n = 6-7, mean ± SD. *P < 0.05, **P < 0.01, ***P < 0.001, ****P < 0.0001. Statistical significance was determined by a 2-tailed Student's *t* test.

**Supplementary Table 1. Antibodies used for immunohistochemistry**

| Antibody name | Company | Catalog No. | Concentration used |
|---|---|---|---|
| Iba1 antibody | Fujifilm | 019-19741 | 1:300 |
| MPO antibody | Invitrogen | SP72 | 1:100 |
| CD4 antibody | Abcam | EPR6855 | 1:100 |
| CD8 antibody | Santa Cruz Biotechnology | sc-1181 | 1:300 |

**Supplementary Table 2. Antibodies used for immunofluorescence staining**

| Antibody name | Company | Catalog No. | Concentration used |
|---|---|---|---|
| Nephrin antibody | Abcam | EPR20993 | 1:300 |
| Synaptopodin antibody | Santa Cruz Biotechnology | sc-515842 | 1:300 |
| Podocin antibody | NOVUSBIO | JB51-33 | 1:300 |
| CD80 antibody | Abcam | EPR1157(2) | 1:200 |
| CD163 antibody | Santa Cruz Biotechnology | sc-58965 | 1:200 |

**Supplementary Table 3: Sequences of the primers used for real-time PCR**

| Primer name | Primer sequence (5'- to -3') | |
|---|---|---|
| RPS18 | F | TTTGCGAGTACTCAACACCAACATC |
| | R | GAGCATATCTTCGGCCCACAC |
| NPHS1 | F | GTCTGCACTGTCGATGCCAATC |
| | R | CCAGTTTGGCATGGTGAATCCG |
| NPHS2 | F | GTGGAAGCTGAGGCACAAAGAC |
| | R | CAGCGACTGAAGAGTGTGCAAG |
| SYNPO | F | CTCTGGTATCCTTTGCCGTCTC |
| | R | TCTGACAGGCTTTCACTCCTCC |
| IL-1β | F | CCACAGACCTTCCAGGAGAATG |
| | R | GTGCAGTTCAGTGATCGTACAGG |
| IL-4 | F | ATCATCGGCATTTTGAACGAGGTC |
| | R | ACCTTGGAAGCCCTACAGACGA |
| IL-6 | F | AGACAGCCACTCACCTCTTCAG |
| | R | TTCTGCCAGTGCCTCTTTGCTG |
| IL-13 | F | AACGGCAGCATGGTATGGAGTG |
| | R | TGGGTCCTGTAGATGGCATTGC |
| TNF | F | CTCTTCTGCCTGCTGCACTTTG |
| | R | ATGGGCTACAGGCTTGTCACTC |
| S100A8 | F | CAAGGAAATCACCATGCCCTCTA |
| | R | ACCATCGCAAGGAACTCCTCGA |
| S100A9 | F | GCACCCAGACACCCTGAACCA |
| | R | TGTGTCCAGGTCCTCCATGATG |

**Supplemental Table 4: Characteristics of participants with and without AD in US adults**

| Characteristics | AD (n = 1488) weighted percentage* | Non-AD (n = 12835) weighted percentage* | *P* value§ |
|---|---|---|---|
| Gender | | | ns |
| Male | 46.03% | 47.64% | |
| Female | 53.97% | 52.36% | |
| Age ± SD (year) | 44.10 ± 15.59 | 43.96 ± 17.68 | ns |
| BMI ± SD ($kg/m^2$) | 28.69 ± 6.97 | 28.49 ± 6.56 | ns |
| AD, atopic dermatitis; SE, standard error; BMI, body mass index; ns, not significant. * Weighted percentage was calculated using NHANES survey design parameters. § P value was calculated using t test for the difference in means of age and BMI, while other tests were $\chi^2$ tests for independence. | | | |
